# Storage Temperature Is More Effective Than Lactic Acid Bacteria Inoculations in Manipulating Fermentation and Bacterial Community Diversity, Co-Occurrence and Functionality of the Whole-Plant Corn Silage

**DOI:** 10.1128/spectrum.00101-22

**Published:** 2022-03-28

**Authors:** Jie Bai, Zitong Ding, Rina Su, Musen Wang, Mengyan Cheng, Dongmei Xie, Xusheng Guo

**Affiliations:** a State Key Laboratory of Grassland Agro-ecosystems, College of Pastoral Agriculture Science and Technology, School of Life Sciences, Lanzhou University, Lanzhou, China; b Probiotics and Biological Feed Research Centre, Lanzhou University, Lanzhou, China; State Key Laboratory of Microbial Resources, Institute of Microbiology, Chinese Academy of Sciences

**Keywords:** *Zea mays*, lactic acid bacteria, bacterial community, bacterial network complexity, function prediction

## Abstract

The objective of this study was to investigate effects of different lactic acid bacteria (LAB) on the fermentation process of whole-plant corn silage stored at different temperatures based on bacterial community successions, interaction networks, and predicted functions. Before ensiling, whole-plant corn was inoculated with *L. plantarum* (LP) or *L. buchneri* (LB) and the silage bags were stored at 20 or 30°C, and sampled after 0.5, 1, 3, 7, 14, and 60 d of ensiling. The higher abundances of *Leuconostoc*, *Pedicoccus* and *Weissella* were observed in silage stored at 30°C after 12 h of ensiling, thereby rapidly decreased pH to about 4.5. According to meta-network analysis, the bacterial communities were more sensitive to storage temperature than LAB inoculants during whole-plant corn ensiling. Species of *Lactobacillus* and *Weissella* were sensitive to 30°C, while *Leuconostoc* species were sensitive to 20°C in whole-plant corn silage. The storage temperature of 30°C decreased bacterial diversity and network complexity of whole-plant corn silage compared with 20°C. Additionally, LP inoculation changed the bacterial community successions during the early and middle ensiling periods, while LB inoculation affected bacterial community successions in the later stage of ensiling. The metabolic pathways of bacterial community were totally different in LB-inoculated silage from that in control and LP-inoculated silage. As the bacterial compositions became simple along with the ensiling process, the functional structure of bacterial community became simplified as well. In general, the storage temperature had a greater impact on the fermentation characteristics, bacterial community and predicted function of whole-plant corn silage compared with LAB inoculations.

**IMPORTANCE** Increased understanding of effects of regulation measures on whole-plant corn silage is important from bacterial community succession, interaction network and predicted functions. According to alpha diversity and meta co-occurrence network, the bacterial communities were more sensitive to storage temperature than LAB inoculants during whole-plant corn ensiling. The storage temperature of 30°C decreased bacterial diversity and network complexity of whole-plant corn silage compared with 20°C. In addition, 30°C promoted the initiation of LP and LB inoculants, and 20°C was conducive to the long-term growth of LP and LB inoculants. According to the changes of bacterial community and predicated functions, it was further confirmed that the effect of LB inoculation was more obvious on whole-plant corn silage.

## INTRODUCTION

Ensiling is a microbial driven process and an appropriate way for preservation of moist forages. Whole-plant corn silage, the main roughage used in the daily ration of ruminants, can be naturally fermented due to its high water-soluble carbohydrate (WSC) content and sufficient epiphytic lactic acid bacteria (LAB) (1). Temperature plays a crucial role in influencing the ensiling process (2, 3). Adesogan (4) reported that hot and humid conditions could be suitable for the growth of some undesirable bacteria and have negative effects on silage fermentation. Generally, the storage temperature from 20 to 30°C is considered suitable for silage fermentation (2). However, there are differences in the optimal storage temperature for ensiling of different types of feed stock, such as 25°C for corn stalk silage (5), 20 to 25°C for whole-plant corn silage (2, 6). According to Liu et al. (7), a weak lactic fermentation was observed in stylo ensiled at 30°C, resulting in a butyric fermentation and protein loss, whereas lactic fermentation was active and dominant when the stylo was ensiled at 20°C.

LAB inoculants, which can alter the fermentation pattern, are widely used to ensure proper fermentation and produce high-quality silage (8). LAB inoculants commonly used in silage are divided into homofermentative (e.g., Lactobacillus plantarum) and heterofermentative culture (e.g., *Latobacillus buchneri*) according to their different fermentation patterns (9, 10). Basically, *L. plantarum* is often used to dominate the lactic acid fermentation and decrease pH consequently, which prevents growth of undesirable microbes and helps to preserve the forage mass (11). *L. buchneri* is used to improve aerobic stability by producing acetic acid and 1, 2-propanediol (9). Previous studies have explored how LAB with different fermentation patterns affected the bacterial communities and regulated the fermentation process of alfalfa and whole-plant corn silage (12, 13). Xu et al. (13) reported that substantial differences in bacterial community compositions and their dynamics were observed between *L. plantarum* and *L. buchneri* inoculated whole-plant corn silages. According to Yang et al. (14), rapid growth of LAB at the initial ensiling stage and subsequently established bacterial community are vital for later fermentation and final silage quality. However, as far as we know, few studies have focused on the effect of LAB with different fermentation patterns on bacterial community successions and functional shifts in whole-plant corn silage stored at different temperatures.

In recent years, the PacBio single molecule in conjunction with real-time sequencing technology (SMRT) has been used to characterize the bacterial community at the species levels in silage (12, 13, 15). The interactions of bacterial communities in silage were also characterized by co-occurrence network (13, 15). It was reported that alfalfa silage with better fermentation quality had simplified bacterial interaction network (15). Additionally, previous studies indicated that the main predicted bacterial functions could explain the feedstock conversion during the composting and ensiling process (15, 16). However, few studies have concentrated on responses of bacterial community successions, their co-occurrences and predicted functions to storage temperature and inoculants during ensiling of whole-crop corn.

Thus, the objectives of this study were to explore effects of two different inoculants (*L. plantarum* and *L. buchneri*) and storage temperatures (20 and 30°C) on fermentation profiles, bacterial community successions, co-occurrences and functional shifts during ensiling of whole-plant corn, and to figure out the responses of bacterial community and their predicated metabolic pathways to ensiling treatments and time.

## RESULTS

### Fermentation and chemical characteristics of whole-plant corn silage.

The fermentation and chemical characteristics of whole-plant corn silage during ensiling are listed in Tables 1 and 2. Overall, there were ensiling time × storage temperature × inoculants interactions (*P < *0.001) for pH, lactic acid and acetic acid concentrations, and lactic acid/acetic acid ratio (Table 1). The pH decreased as early as 12 h of ensiling in the whole-plant corn silage stored at 30°C (*P < *0.001). There was no difference in pH between control and LP-inoculated silage after 60 of ensiling, whereas LB-inoculated silage had a higher pH than control and LP-inoculated silage. As expected, the acetic acid concentration was higher in LB-inoculated silage than control and LP-inoculated silage. Propionic acid of whole-plant corn silage was only detected after 60 d of ensiling, and the LB inoculation increased propionic acid concentration compared with control and LP-inoculated silage. It is worth noting that higher levels of lactic and propionic acid were observed in whole-plant corn silage stored at 30°C versus 20°C, whereas acetic acid was higher in silage stored at 20°C.

**TABLE 1:** Fermentation characteristics of whole-plant corn silage inoculated with *L. plantarum* and *L. buchneri* stored at different temperatures during ensiling^*a*^

Item	D	20°C			30°C			SEM^*b*^	*P* value						
		CK	LP	LB	CK	LP	LB		D	T	I	D × T	D × I	T × I	D × T×I
pH	0.5	5.95	5.87	6.02	4.51	4.41	4.49	0.001	<0.001	<0.001	<0.001	<0.001	<0.001	<0.001	<0.001
	1	4.99	4.89	4.93	4.15	3.97	4.03								
	3	4.05	3.91	4.01	3.75	3.81	3.76								
	7	3.8	3.79	3.81	3.74	3.77	3.76								
	14	3.8	3.77	3.79	3.76	3.78	3.81								
	60	3.71	3.71	3.81	3.58	3.57	3.65								
	avg	4.38	4.32	4.39	3.92	3.89	3.92								
Lactic acid (g/kg DM)	0.5	9.24	14.4	9.57	18.5	21.2	15.5	0.132	<0.001	<0.001	<0.001	<0.001	<0.001	<0.001	<0.001
	1	16.6	19. 8	17.3	28.7	35.9	30.1								
	3	33.1	41. 8	33.5	63.4	77.4	67								
	7	55.6	57.2	44.1	65.8	80.7	75.4								
	14	57.9	73.7	61.0	56.7	75.9	60. 7								
	60	85.3	94.0	77.1	97.0	93.7	84.2								
	avg	43.0	50.1	40.4	55.0	64.1	55.5								
Acetic acid (g/kg DM)	0.5	N	N	N	N	N	N	0.036	<0.001	<0.001	<0.001	<0.001	<0.001	<0.001	<0.001
	1	1.15	1.88	3.01	6.05	5.4	6.61								
	3	8.36	7.65	8.56	7.88	10.7	12.7								
	7	13.0	10.9	15.2	7.74	5.92	12.1								
	14	12.3	9.57	12.9	8.57	10.2	16.5								
	60	12.1	14.9	26.8	10.3	9.00	15.5								
	avg	7.82	7.48	11.07	6.76	6.87	10.6								
Propionic acid (g/kg DM)	0.5	N	N	N	N	N	N	0.044	N	<0.001	<0.001	N	N	<0.001	N
	1	N	N	N	N	N	N								
	3	N	N	N	N	N	N								
	7	N	N	N	N	N	N								
	14	N	N	N	N	N	N								
	60	3.28	3.61	5.14	6.12	5.09	7.53								
	avg	N	N	N	N	N	N								
Lactic acid/acetic acid	0.5	N	N	N	N	N	N	0.02	<0.001	<0.001	<0.001	<0.001	<0.001	<0.001	<0.001
	1	3.96	4.96	2.96	4.34	6.48	4.54								
	3	4.06	5.2	4.28	7.56	7.19	5.42								
	7	5.87	6.02	5.63	6.75	7.78	4.58								
	14	5.73	6.31	4.73	6.44	7.46	3.64								
	60	6.48	6.63	2.83	10.12	10.63	6.3								
	avg	4.35	4.86	3.4	5.87	6.59	4.08								

a CK, control, no inoculations; LP, silages inoculated with Lactobacillus plantarum MTD/1; LB, silages inoculated with Lactobacillus buchneri 40788. DM, dry matter; D, ensiling days; T, storage temperatures; I, inoculants; D × T, interaction between ensiling days and storage temperatures; D × I, interaction between ensiling days and inoculants; T × I, interaction between storage temperatures and inoculants; D × T×I, interaction among ensiling days, temperatures and inoculants; N, not detected.

b SEM, standard error of the mean.

**TABLE 2:** Chemical compositions of whole-crop corn silages inoculated with *L. plantarum* and *L. buchneri* stored at different temperatures for 60 d

Items^*a*^	Temperatures	Inoculants^*b*^				*P*-value^*d*^		
		CK	LP	LB	SEM^*c*^	I	T	I × T
DM, g/kg FM	20°C	271	273	264	0.494	<0.001	<0.001	0.555
	30°C	282	281	275				
WSC, g/kg DM	20°C	97.3	98.0	97.4	0.427	0.002	<0.001	0.001
	30°C	119	110	109				
CP, g/kg DM	20°C	89.7	91.0	91.0	0.195	0.023	<0.001	0.977
	30°C	84.9	86.1	86.3				
NPN, g/kg TN	20°C	276	227	225	0.896	<0.001	<0.001	0.437
	30°C	327	285	280				
NH_3_-N, g/kg TN	20°C	67.5	65.5	65.7	0.179	0.003	<0.001	0.28
	30°C	76.6	74.9	76.2				
aNDF, g/kg DM	20°C	48.7	47.6	48.8	0.087	<0.001	0.001	<0.001
	30°C	50.2	49.3	47.8				
ADF, g/kg DM	20°C	24.1	24.2	26.1	0.127	0.019	0.023	0.008
	30°C	25.3	25.7	25.3				

a DM, dry matter; FW, fresh weight; WSC, water soluble carbohydrates; CP, crude protein; TN, total nitrogen; NPN, nonprotein nitrogen; NH_3_-N, ammonia N; aNDF, neutral detergent fiber assayed with a heat stable amylase and expressed inclusive of residual ash; ADF, acid detergent fiber expressed inclusive of residual ash.

b CK, control, no inoculations; LP, silages inoculated with Lactobacillus plantarum MTD/1; LB, silages inoculated with Lactobacillus buchneri 40788.

c SEM, standard error of the mean.

d I, inoculants; T, storage temperatures; I × T, interaction between inoculants and storage temperatures.

There were inoculants × storage temperature interactions (*P < *0.05) for concentrations of silage WSC, neutral detergent fiber (aNDF) and acid detergent fiber (ADF) after 60 d of ensiling (Table 2). The LP and LB inoculations decreased the WSC content of whole-plant corn silage stored at 30°C, while there was no difference in WSC content among the three silage groups stored at 20°C. The increased ADF content was observed in LB-inoculated silage stored at 20°C compared with control and LP-inoculated silage, while no difference was observed on ADF among the three groups ensiled at 30°C. In addition, silage stored at 30°C had higher DM, nonprotein nitrogen (NPN) and ammonia nitrogen (NH_3_-N), and lower crude protein (CP) than that stored at 20°C.

### Bacterial community diversity, compositions, and successions in whole-plant corn silage.

**FIG 1:**
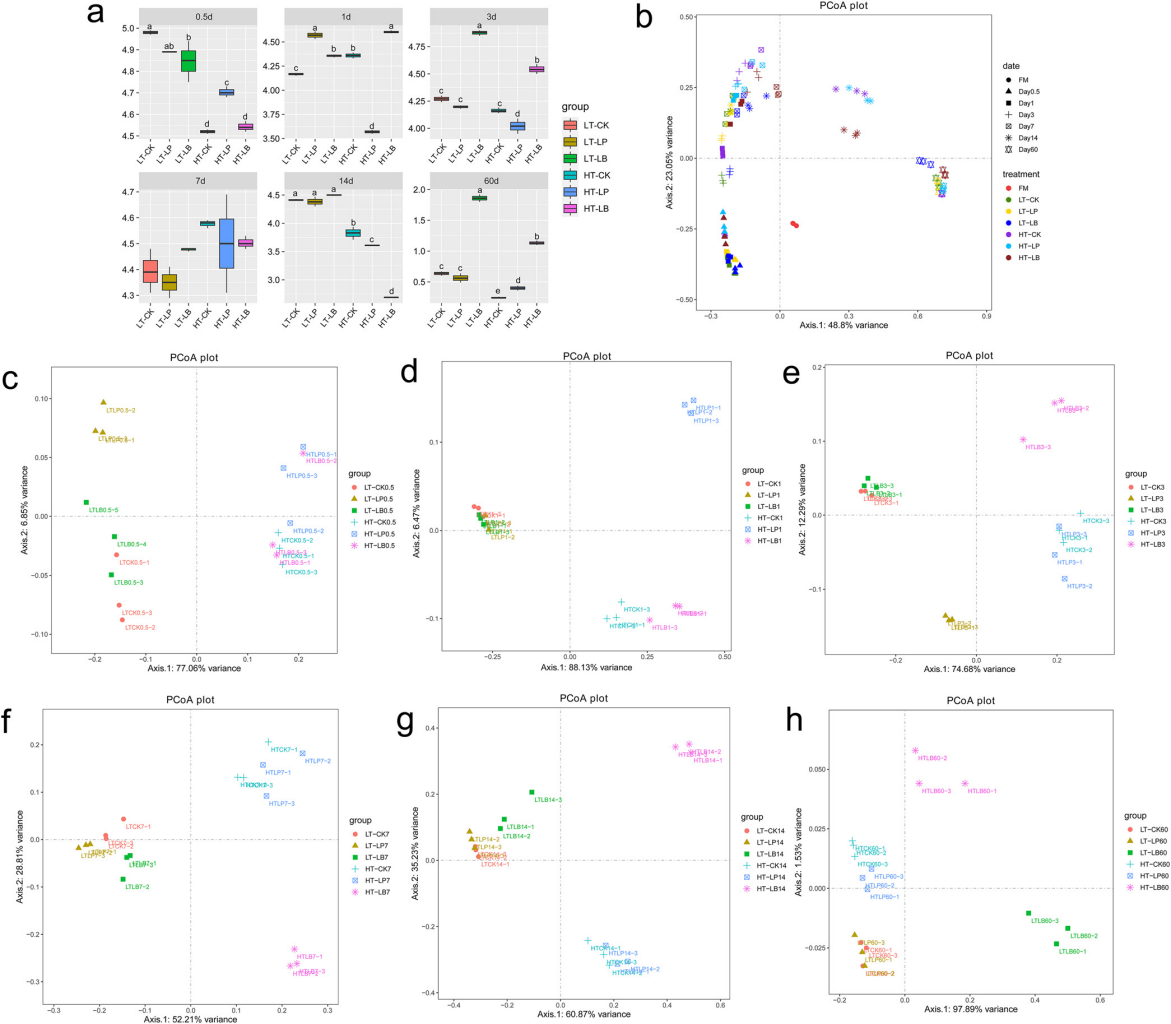
Bacterial community diversities of whole-plant corn silage during ensiling. CK, Control (samples without inoculants); LP, silages inoculated with Lactobacillus plantarum MTD/1; LB, silages inoculated with Lactobacillus buchneri 40788. LT, silage stored at 20°C; HT, silage stored at 30°C. Arabic number indicating days of ensiling. (a) The variations in community alpha-diversities (Shannon index). (b) The community dissimilarities in different groups and fermentation times, calculated using Principal Coordinates Analysis (PCoA). (c–h) PCoA analysis after 0.5, 1, 3, 7, 14, and 60 d of ensiling, respectively.

**FIG 2:**
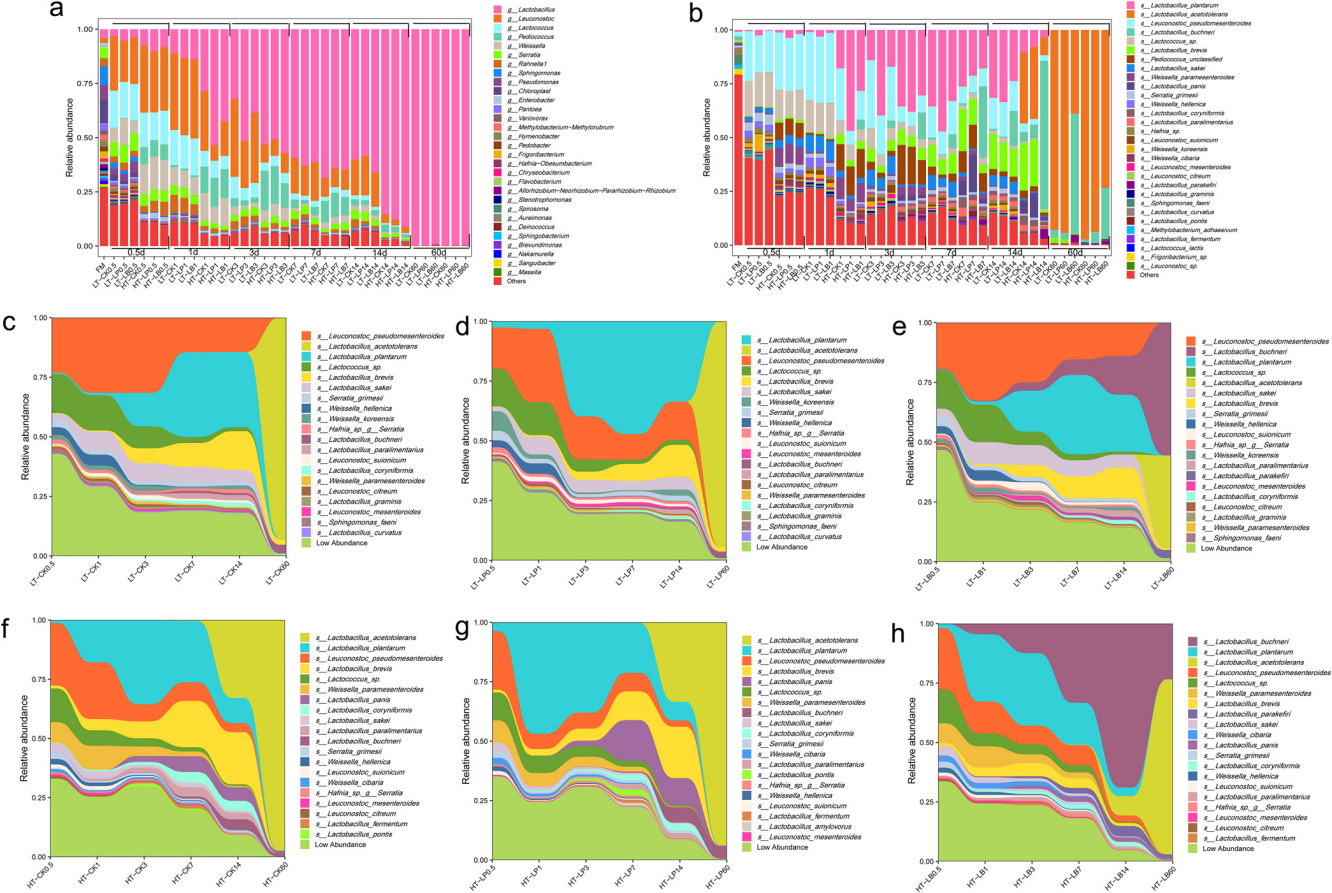
Bacterial community compositions and successions of whole-plant corn silage during ensiling. CK, Control (samples without inoculants); LP, silages inoculated with Lactobacillus plantarum MTD/1; LB, silages inoculated with Lactobacillus buchneri 40788. LT, silage stored at 20°C; HT, silage stored at 30°C. Arabic number indicating days of ensiling. (a) Relative abundances of whole-plant corn silage bacterial genera across different groups and fermentation times. (b) Relative abundances of whole-plant corn silage bacterial species across different groups and fermentation times. Bacterial community successions of whole-plant corn silage are aggregated and colored by species on a stream-graph. (c) The control stored at 20°C; (d) LP-inoculated silage stored at 20°C; (e) LB-inoculated silage stored at 20°C; (f) the control stored at 30°C; (g) LP-inoculated silage stored at 30°C; (h) LB-inoculated silage stored at 30°C.

Bacterial diversity, community composition, and succession of whole-plant corn silage are shown in Fig. 1 and 2, respectively. In general, the alpha diversity (Shannon index) was lower in silage stored at 30°C than 20°C during ensiling (Fig. 1a). According to the principal coordinates analysis based on bray curtis (PCoA, beta diversity), significant differences and regular changes in bacterial communities of whole-plant corn silage at different fermentation periods were observed (Fig. 1b). According to the beta diversity at each ensiling time (Fig. 1c to h), the bacterial communities of whole-plant corn silage stored at 20°C were clearly separated from that stored at 30°C by the axis 1 after 0.5, 1, 3, 7 and 14 d of ensiling, and b*y* axis 2 after 60 d of ensiling. When the whole-plant corn silage stored at 30°C, the bacterial communities of LB-inoculated silage were clearly separated from control and LP-inoculated silage after 3, 7, 14, and 60 d of ensiling (Fig. 1e to h). While the bacterial community of LB-inoculated silage was clearly separated from control and LP-inoculated silages only after 60 d of ensiling when stored at 20°C.

The bacterial community compositions of whole-plant corn silage at genus and species levels are shown in Fig. 2a and b, respectively. The bacterial community composition of fresh corn material was complex before ensiling. After ensiling, *Leuconostoc*, *Lactococcus*, *Peidococcus*, and *Weissella* were gradually replaced by *Lactobacillus*, and *Lactobacillus* became the dominant genera after 60 d of ensiling (Fig. 2a). The storage temperature obviously affected bacterial community compositions of whole-plant corn silage. The relative abundances of *Pediococcus* and *Weissella* were higher in silage stored at 30°C than 20°C during the entire ensiling period, whereas the relative abundance of *Leuconostoc* was higher in silage stored at 20°C than 30°C. When the bacterial information was annotated to the species levels, differences were observed in bacterial community compositions between control and LAB-inoculated silages (Fig. 2b). When the silage stored at 30°C, the relative abundance of *L. plantarum* rapidly increased in LP-inoculated silage compared with control after 1 d of ensiling, while it decreased from 7 to 14 d of ensiling along with the increased relative abundance of Lactobacillus Panis. The relative abundance of *L. buchneri* gradually increased in LB-inoculated silage stored at 30°C and always higher than that in control and LP-inoculated silage from 1 to 60 d of ensiling. When the silage stored at 20°C, *L. plantarum* and *L. buchneri* abundances were higher in LP- and LB-inoculated silages than control from 3 to 14 d of ensiling, respectively. After 60 d of ensiling, the bacterial community compositions became simple and the dominant bacteria species was *L. acetotolerans* except for LB-inoculated silage stored at 20°C, in which the dominant species was *L. buchneri*.

According to the stream-graph (Fig. 2c to h), both inoculants and storage temperature had a remarkable influence on the succession of bacterial communities in whole-plant corn silage. The LP inoculation affected the bacterial community succession from 0.5 to 14 d of ensiling (Fig. 2d and g), while LB inoculation had a significant impact on the bacterial community succession in the later stage of ensiling (14 to 60 d of ensiling) (Fig. 2e and h). The storage temperature had a remarkable effect on the dynamics of bacterial community in whole-plant corn silage. The temperature of 30°C accelerated the occupations of *L. plantarum*, *L. buchneri* and *L. acetotolerans* in the bacterial community of silage.

### Bacterial co-occurrence, co-occurrence network complexity, and stability in whole-plant corn silage.

**FIG 3:**
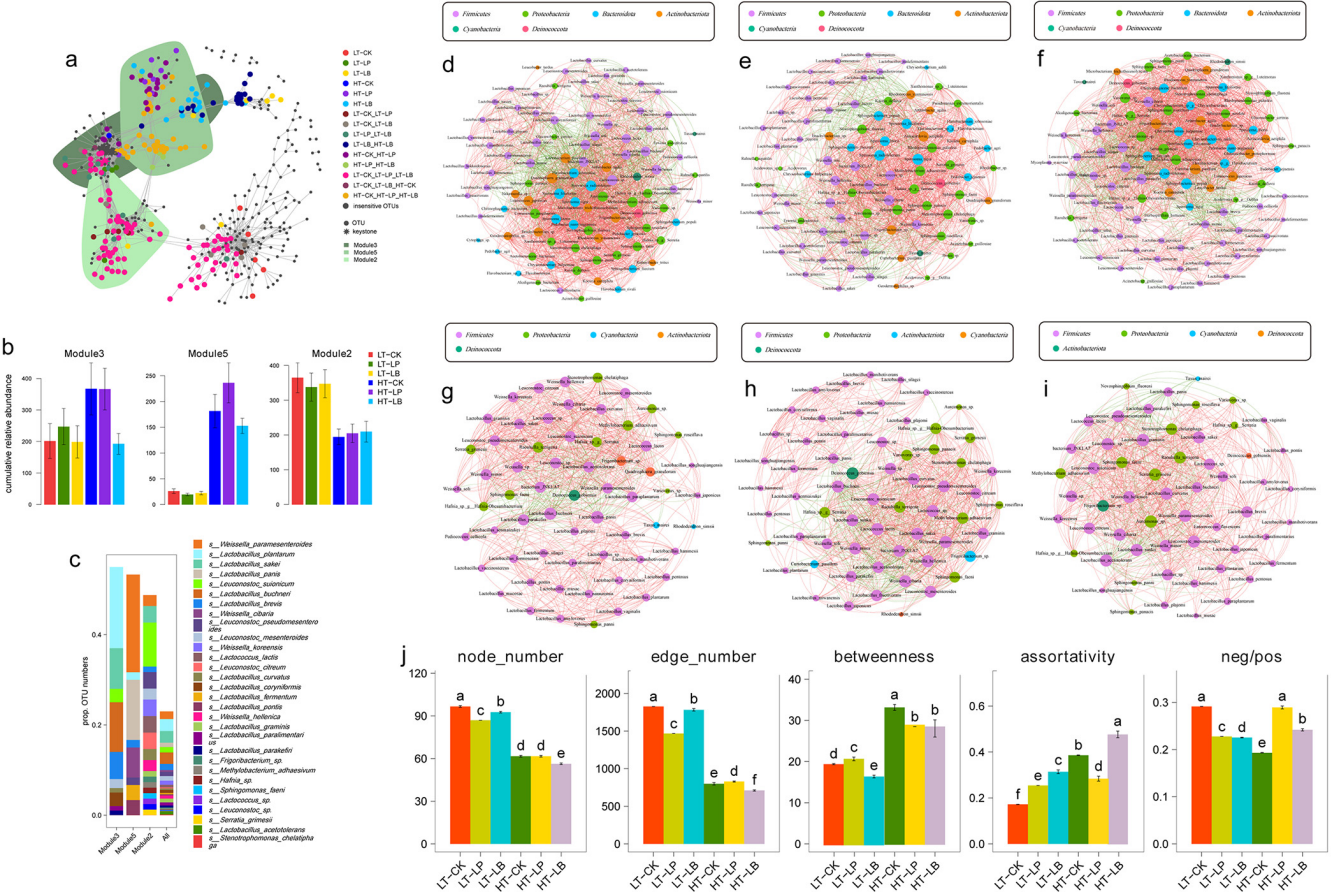
Co-occurrence patterns in whole-plant corn silage bacterial as affected by inoculants and storage temperature. (a) Meta co-occurrence patterns of storage temperature and inoculants sensitive OTUs. Co-occurrence networks visualizing significant correlations (*P* < 0.001, indicated with gray lines) between bacteria. OTUs are colored by their association to the different inoculants and different storage temperatures, gray OTUs are insensitive to inoculants and storage temperatures. Shaded areas represent the network modules containing OTUs sensitive to inoculants and storage temperatures. (b) Cumulative relative abundance (as counts per million) of all bacteria of the inoculants and storage temperature sensitive modules in whole-plant corn silage. The cumulative relative abundance in silages of LT-CK (red), LT-LP (green), LT-LB (yellow), HT-CK (blue), HT-LP (purple), HT-LB (light blue) indicates the overall response of inoculants and storage temperature sensitive modules to the silage regulations. (c) Qualitative taxonomic composition of inoculants and storage temperatures sensitive modules is reported as proportional OTUs numbers per bacterial species. d-i. Co-occurrence network of silage bacterial in (d) the control stored at 20°C, (e) LP-inoculated silage stored at 20°C, (f) LB-inoculated silage stored at 20°C; (g) the control stored at 30°C; (h) LP-inoculated silage stored at 30°C; (i) LB-inoculated silage stored at 30°C. (j) The numbers of node and edge and the degree of betweenness and assortativity of silage bacteria co-occurrence patterns. Neg/pos, the ratio of negative correlation and positive correlation. Means with the same lower case were not significant at *P* < 0.05 among the silage groups.

The distribution patterns of inoculants and storage temperature sensitive OTUs in meta-network of bacterial communities are shown in Fig. 3a to c. It is noted that three modules (module 3, 5 and 2) contained relatively high proportions of sensitive OTUs (Fig. 3a). The relative abundances of these module members (Fig. 3b) and their distributions in the network reflected that the dissimilarity of bacterial communities was mainly driven by temperature. The effect of storage temperature in bacterial community was clearly reflected by the discrete module 2 and module 5 in the network. The sensitive OTUs specific to 20°C and 30°C were, respectively, clustered together into module 2 and module 5. Module 3 primarily contained sensitive OTUs specific to control and LP-inoculated silage stored at 30°C. Furthermore, the temperature responsive modules were comprised of different bacteria species. Module 5 mainly contained Weissella paramesenteroides, *L. panis*, *W. cibaria*, *L. fermentum*, and *L. pontis*, whereas *Leuconostoc* species were the major LAB bacteria in module 2 (Fig. 3c).

The networks of bacterial communities in whole-plant corn silage inoculated with the LP or LB strain stored at different temperatures demonstrated distinct co-occurrence patterns (Fig. 3d to i). The network topological parameters of node and edge numbers, degrees of betweenness and assortativity were used to assess the bacterial network complexity, with higher node and edge numbers and lower betweenness and assortativity representing greater network complexity. The ratios of negative and positive correlations (neg/pos) were used to assess the bacterial network stability, with higher neg/pos ratio representing greater network stability (Fig. 3j). The results showed that storage temperature had more obvious effect on bacterial associations than inoculants used in the present study. Regardless of whether the LAB inoculants were inoculated or not, the storage temperature of 30°C dramatically reduced the complexity of bacterial community network compared with 20°C. Interestingly, LP and LB inoculations decreased the bacterial community stability (neg/pos ratio) of whole-plant corn silage stored at 20°C, while a contrary result was observed when the silage stored at 30°C. Meanwhile, the bacterial community stability was higher in LP-inoculated silage than LB-inoculated silage regardless of storage temperature.

### Predicted functions of bacterial communities during whole-plant corn ensiling.

**FIG 4:**
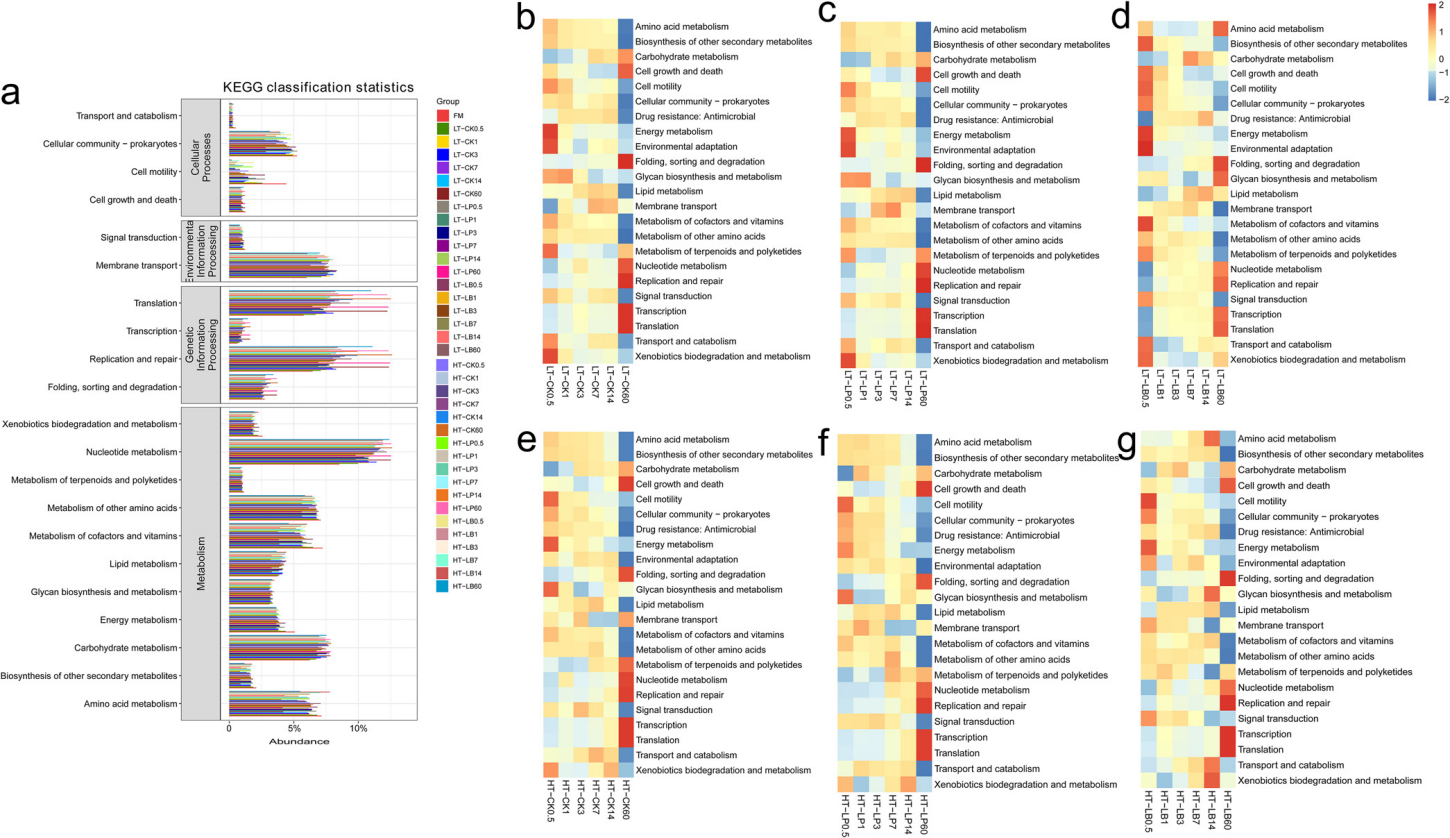
Bacterial alterations that contribute to functional shifts after fermentation in whole-plant corn silage. LB, silages inoculated with Lactobacillus buchneri 40788. LT, silage stored at 20°C; HT, silage stored at 30°C. Arabic number indicating days of ensiling. Summary of functional shifts predicted using Phylogenetic Investigation of Communities by Reconstruction of Unobserved States (PICRUSt2). For each KEGG pathway, the second level of the predicted functional shift is shown with respect to the different groups and fermentation processes. (a) Level 2 KEGG orthologue functional predictions explained by PICRUSt2. Comparing taxon-level contribution profiles of functional shifts in fermentation process by FishTaco approach. (b) the control stored at 20°C; (c) LP-inoculated silage stored at 20°C; (d) LB-inoculated silage stored at 20°C; (e) the control stored at 30°C; (f) LP-inoculated silage stored at 30°C; (g) LB-inoculated silage stored at 30°C.

In comparison with control, LP and LB inoculations modulated the bacterial communities of whole-plant corn silage, which contributed to striking differences in predicated functions with the extension of ensiling time (Fig. 4a to g). Among the predicted functions, remarkable upregulations in functions of transcription, translation, and replication and repair were observed in 60 d silages compared with other ensiling times (Fig. 4a). The functional abundance heat-map shows the functional shifts of bacterial communities in whole-plant corn silage with the extension of ensiling time (Fig. 4b to g). When silage stored at 20°C, the carbohydrate metabolism was upregulated in control after 7 d of ensiling, whereas it was upregulated in LAB-inoculated silage after 3 d of ensiling. While when silage stored at 30°C, the carbohydrate metabolism was upregulated in control after 3 d of ensiling, whereas it was upregulated in LAB-inoculated silage after 1 d of ensiling. The amino acid metabolism was downregulated gradually in control and LP-inoculated silage stored at the two temperatures, whereas it was downregulated in LB-inoculated silage stored at 20°C in early stage (1 to 7 d) of ensiling, and then upregulated in middle and late stage (14 to 60 d) of ensiling. When the LB-inoculated silage stored at 30°C, amino acid metabolism was upregulated from 0.5 to 14 d, while it was downregulated after 60 d of ensiling.

**FIG 5:**
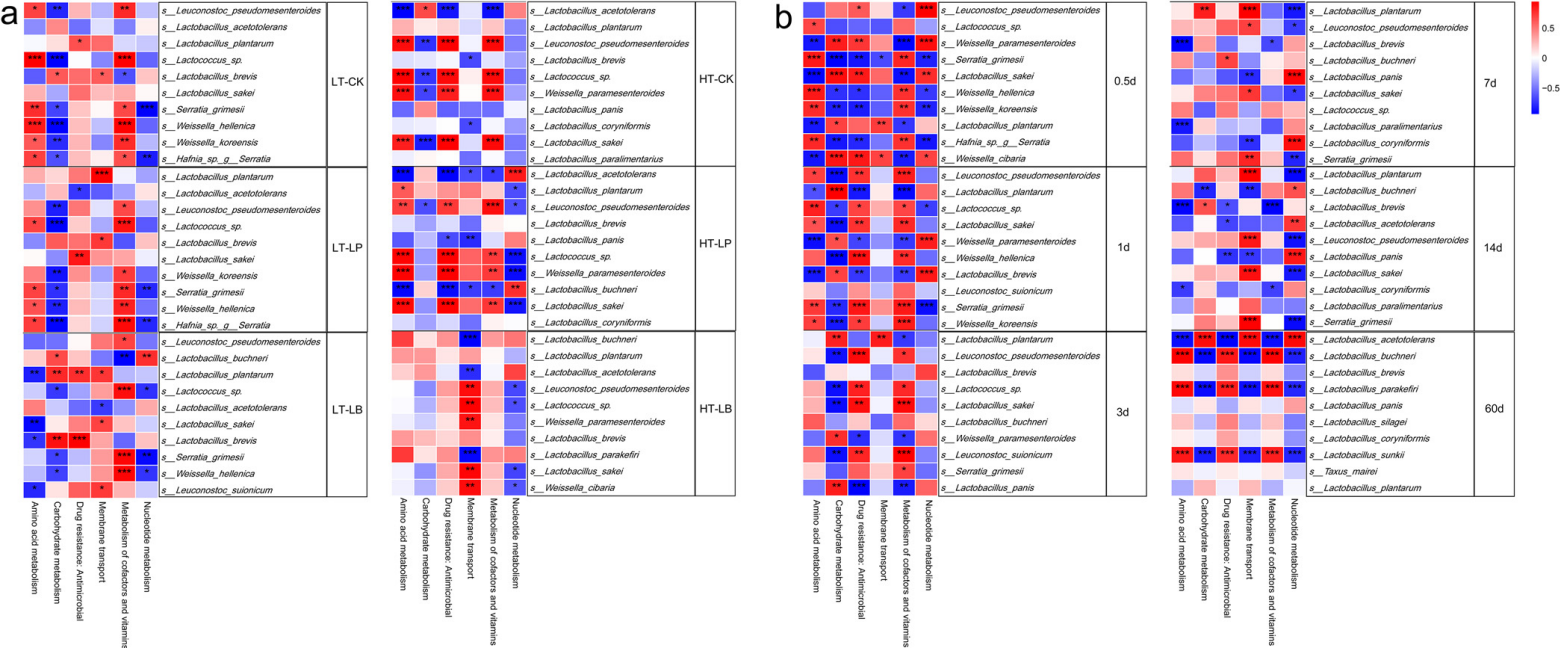
Association analysis between bacterial species and metabolism pathways. Metabolism pathways are displayed horizontally, and the bacterial species are displayed vertically. The corresponding value of the middle heat map is the Spearman correlation coefficient r, with ranges between −1 and 1; *r* < 0 indicates a negative correlation (blue), *r* > 0 indicates a positive correlation (red), and “*”, “**”, and “***” represent *P* < 0.05, *P* < 0.01, and *P* < 0.001, respectively. (a) Correlations between bacterial species and metabolic pathways in different silage groups. CK, Control (samples without inoculants); LP, silages inoculated with Lactobacillus plantarum MTD/1; LB, silages inoculated with Lactobacillus buchneri 40788. LT, silage stored at 20°C; HT, silage stored at 30°C. (b) Correlations between bacterial species and metabolic pathways at different ensiling time points (after 0.5, 1, 3, 7, 14, and 60 d of ensiling).

In order to explore the contribution of bacteria to metabolic pathways during ensiling process, a correlation analysis between the main predicated functions and the top10 bacterial species was performed (Fig. 5). The bacterial species that positively correlated with amino acid metabolism were also positively related to metabolism of cofactors and vitamins in control and LP-inoculated silages stored at both temperatures (Fig. 5a). The LP inoculation had a little effect on the correlations between bacterial metabolism pathways and bacterial species compared with control, while LB inoculated completely changed the bacterial species that contributed to metabolic pathways. In addition, *L. plantarum* was positively correlated with membrane transport in LP-inoculated silage stored at 20°C, while it was positively correlated with amino acid metabolism and negatively correlated with nucleotide metabolism in LP-inoculated silage stored at 30°C. *L. buchneri* was positively correlated with carbohydrate metabolism and nucleotide metabolism but negatively correlated with metabolism of cofactors and vitamins in LB-inoculated silage stored at 20°C, while *L. buchneri* was negatively correlated with membrane transport in LB-inoculated silage stored at 30°C.In addition, the influence of bacterial species on metabolism pathways with the extension of ensiling time was explored (Fig. 5b). The correlations between bacterial species and metabolic pathway were more complicated when the silage fermentation initiated, and the correlations became simple after 3 d of ensiling.

## DISCUSSION

The optimum temperature for silage fermentation and microbial activity is between 20 and 30°C (2). The present study showed that lactic acid fermentation of whole-plant corn silage was promoted by the storage temperature of 30°C versus 20°C. According to the metabolic theory of ecology, microbial metabolism is usually more active in a warmer environment (17, 18). However, the higher acetic acid concentration in corn silage stored at 20°C after 60 d of fermentation indicated that heterofermentative LAB might be more suitable for relatively low storage temperature. In addition, both LP and LB inoculations increased lactic and acetic acid concentrations of corn silage. Xu et al. (19) also reported that LB inoculation increased acetic acid concentration and pH of whole-plant corn silage after ensiling 90 d. In addition, the LB inoculation increased propionic acid concentration in whole-plant corn silage. Driehuis et al. (20) reported that silage micrrorganisms could further degrade 1, 2-propanediol to propionic acid and 1-propanol, resulting in the accumulation of propionic acid in inoculated silages. Additionally, Elferink et al. (9) reported that the LB inoculation improved aerobic stability by fermenting lactic acid to acetic acid and 1, 2-propanediol. Therefore, the increased concentration of propionic acid in LB inoculated silage might be due to production of 1, 2-propanediol by LB.

Generally, silage with high fermentation quality has low alpha diversity (15). In the present study, the deceased Shannon index in whole-plant corn silage stored at 30°C compared with 20°C during ensiling was consistent with the rapid lactic acid fermentation in silage stored at 30°C. According to Bai et al. (15), the decreased alpha diversity was observed in Pediococcus pentosaceus inoculated alfalfa silage after 3 d of ensiling, which had the lowest pH and most acidic silage environment. According to the PCoA analysis, the bacterial diversity of whole-plant corn silage stored at 20°C were clearly separated from that stored at 30°C during the ensiling stages from 0.5 to 60 d. It seems that LAB initiated the fermentation of whole-plant corn silage as early as 12 h of ensiling, and the storage temperature obviously affected bacterial community of whole-plant corn silage after 12 h of ensiling. This result was in accordance with the rapidly decreased pH in silage stored at 30°C after 12 h of ensiling. It was worth noting that the bacterial diversity was distinguishable between LB-inoculated silage and control or LP-inoculated silage when stored at 30°C from 3 to 60 d of ensiling, which was inconsistent with the report of Xu et al. (13), in which *L. plantarum* and *L. buchneri* inoculations did not clearly separate bacterial diversity of whole-crop corn silage compared with control. In addition, the LB-inoculated silage was clearly separated from control and LP-inoculated silage when stored at 20°C after ensiling 60 d, which might be explained by the high pH in LB-inoculated silage fermented for 60 d. However, the control was not separated from LP-inoculated silage stored at the two temperatures, indicating that the effect of LB inoculants on the bacterial community was more obvious than LP inoculants in whole-plant corn silage, especially when the silage stored at 30°C.

At the initiating fermentation stage of 0.5 d, the appearance of *Pediococcus* and increased abundances of *Leuconostoc* and *Weissella* in silage stored at 30°C versus 20°C was consistent with the rapidly decreased pH in whole-plant corn silage stored at 30°C. As Ni et al. (21) and Yang et al. (14) reported that *Pediococcus*, *Leuconostoc* and *Weissella*, as lactic acid-producing LAB, initiated lactic acid fermentation at the early ensiling periods. During the ensiling period from 1 to 14 d, the higher relative abundance of *Leuconostoc* in silage stored at 20°C versus 30°C and the higher *Pediococcus* in silage stored at 30°C versus 20°C indicated that *Leuconostoc* grow vigorously in whole-plant corn silage in a storage environment of 20°C, while *Pediococcus* was more suitable to grow in silage stored at 30°C.

According to Wang and Nishino (22), *L. panis* has never been isolated from crop silage; thus, the effect of this LAB species on whole-plant corn silage is unclear. Meroth et al. (23) found that *L. panis* was dominant in sourdough prepared at 30 and 40°C. The appearance of *L. panis* in whole-plant corn silage stored at 30°C after 3 d of ensiling and its rapid increase in LP-inoculated silage from 7 to 14 d of ensiling indicated that *L. panis* was also suitable to grow in whole-plant corn silage stored at a relative high temperature, and LP inoculation accelerated its growth in whole-plant corn silage. After 60 d of ensiling, *L. acetotolerans* became the dominant bacteria in control and LP-inoculated silage. This species was also identified as a dominant species in whole-plant corn silage during the later ensiling periods in the report of Xu et al. (13). *L. acetotolerans*, an acetic acid-tolerant LAB, was first isolated from spoiled broth of rice vinegar by Entani et al. (24), which is facultatively anaerobic species and produces DL-lactic acid homofermentatively. This might explain the increased LA/AA ratios in control and LP-inoculated silage compared with LB-inoculated silage. Based on the stream graphs of bacterial community dynamics, the storage temperature completely changed bacterial community successions in whole-plant corn silage. The storage temperature of 30°C promoted the initiated fermentation of *L. plantarum* and *L. buchneri* in LP- and LB-inoculated silage, respectively. This might be explained by the rapid growth of LAB in an environment of 27∼38°C (25). Meanwhile, the present results further confirmed that LP inoculants were more competitive during the early stage of ensiling and LB inoculants grew more vigorously at the later ensiling stage.

In the meta-networks of whole-plant corn silage bacteria, the sensitive OTUs grouped in distinct modules, which reflected the stronger effect of storage temperature rather than inoculants on the bacterial community in whole-plant corn silage. In addition, the temperature responsive modules were comprised of different bacteria, revealing that different storage temperature targeted different microbial lineages. Yamamoto et al. (25) reported that the environment of 27∼38°C was more suitable for rapid growth of LAB. However, few studies have reported the influence of temperature on the growth of different LAB species during ensiling. The present meta-network analysis results showed that *Lactobacillus* and *Weissella* species were sensitive to the storage temperature of 30°C, whereas *Leuconostoc* species were sensitive to 20°C during whole-plant corn ensiling. Additionally, the bacterial compositions in modules 3 and 5 that responded to the storage temperature of 30°C were relatively simple, while the bacterial composition in the module 2 responded to 20°C was more complex, which was consistent with the results on alpha diversity.

The separate bacterial network was used to further explore the effect of LP and LB inoculations on bacterial network complexity and stability of whole-plant corn silage stored at different temperatures. The results further demonstrated that the storage temperature had a greater impact on bacterial network complexity than LAB inoculations. In our previous study, we found that P. pentosaceus or *L. plantarum* inoculated alfalfa silage with simple bacterial interaction network had a high fermentation quality (15). In this study, high storage temperature reduced the bacterial network complexity in whole-plant corn silage, which was in accordance with the rapid fermentation initiation and the decreased alpha diversity in silage stored at 30°C. It might be due to the inhibition of undesirable microorganism growth by the rapid initiation of lactic acid fermentation and reduced pH in corn silage stored at 30°C. Fan et al. (26) reported that negative interactions might weaken competitive relations of bacterial community, whereas positive interactions could strengthen competitive relations. Therefore, higher neg/pos ratio represents weaker competition between microbial communities and more stable microbial network structure. In the present study, the storage temperature of 30°C decreased the bacterial network stability of the whole-plant corn silage without LAB inoculations compared with 20°C. As predicated by the metabolic theory of ecology (MTE) (27), rising temperature would stimulate various biotic interactions (predation, parasitism, competition and symbiosis) because of more active in individual metabolic process and faster growth normally occurred at higher temperature. As a result, the stronger activity and competition of epiphytic bacteria reduced the bacterial network stability of whole-plant corn silage stored at 30°C. However, LP and LB inoculations increased bacterial network stability of corn silage stored at 30°C compared with 20°C. The reason is unclear. Interestingly, we found the bacterial network complexity was decreased in LP-inoculated silage stored at 20°C compared with control, whereas that was increased in silage stored at 30°C. This result indicated that the LP inoculation not only affected the bacterial network complexity but also influenced the network stability.

Silage fermentation process of is an organic acids accumulation process (28). The protonated acid may enter the cells and then dissociate into proton and corresponding ion, which leads to the increase in intracellular acidity and accelerates the metabolic disorders of the cells (29, 30). In the present study, the functions of transcription, translation, and replication and repair were upregulated remarkably in 60 d silages compared with other ensiling times. Therefore, these upregulated genetic functions were likely attributed to the responses of microorganisms to the long-term acid stress in silage. Translation is a process in which mRNA is used as a template and tRNA is used as a vehicle to assemble activated amino acids on the ribosome into a protein polypeptide chain under the action of related enzymes, cofactors and energy. The amino acid metabolism in this study was obviously downregulated in 60 d silages, which was consistent with the upregulation of translation. The carbohydrate metabolism is a most important metabolism pathway in silage process whereby LAB converts WSC into organic acids. In the present study, the storage temperature of 30°C and LAB inoculation advanced the time of upregulation of carbohydrate metabolism, which was consistent with the rapidly initiated fermentation and increased lactic acid concentration in silage stored at 30°C as early as 0.5 d of ensiling. Meanwhile, LP and LB inoculations in silages increased lactic acid and acetic acid concentration, respectively, which was also related to the preferential upregulation of carbohydrate metabolism in this study. Novik and Savich (31) reported that LAB did not synthesize all their essential amino acids, and they relied on proteolytic systems to provide essential amino acids for their growth. Consequently, the observed dynamic of amino acid metabolism in the three groups stored at different temperatures might reflect the metabolism in the dominant population throughout the ensiling process. In the present study, the LB inoculation modulated the bacterial communities during ensiling process, which resulted in marked differences in amino acid metabolism shift. Indeed, according to the correlations between the amino acid metabolism and bacterial species, the bacterial species contributed to amino acid metabolism changed completely in LB-inoculated silage compared with control and LP-inoculated silage.

### Conclusions.

The storage temperature of 30°C improved the growth of lactic acid-producing LAB such as *Leuconostoc*, *Pediococcus* and *Weissella* of whole-plant corn silage, thereby reduced silage pH to about 4.5 as rapidly as 12 h of ensiling. Meanwhile, the storage temperature of 30°C changed bacterial community successions of whole-plant corn silage, and decreased bacterial diversity and network complexity compared with the silage stored at 20°C. The growth and fermentation speed of LP and LB inoculants was also faster in whole-plant corn silage stored at 30°C than 20°C. The LP inoculation had an impact on bacterial community successions during the early and middle ensiling periods, and the dominant bacteria was *L. acetotolerans* in control and LP-inoculated silage after 60 d of ensiling. In contrast, LB inoculation mainly changed bacterial community successions in the later stages of ensiling and *L. acetotolerans* and *L. buchneri* were the dominant bacteria. The effects of storage temperature on the fermentation quality, bacterial community and predicted function of whole-plant corn silage were greater than LP and LB inoculations. In the bacterial communities of present silage, the LAB species of *Lactobacillus* and *Weissella* were sensitive to 30°C, whereas *Leuconostoc* species were sensitive to 20°C. Additionally, it was further confirmed that the effect of LB inoculation was more obvious through the changes of bacterial community and predicated functions. In general, we recommend choosing warmer weather for silage production in actual production, and choosing LB as inoculants for whole-plant corn silage.

## MATERIALS AND METHODS

### Silage preparation.

Whole-plant corn material (Zea mays *L.* Dajingjiu3876) was harvested at half milk-line from 3 randomly selected sites (used as replication for each treatment) within a field of about 0.1 ha in a farm located in Anding County, Dingxi city, Gansu province, China. The DM content of the fresh whole-plant corn was 277 g/kg of fresh weight, and contents of WSC, CP, aNDF and ADF were 324, 91.1, 51.0 and 25.4 g/kg DM, respectively. The whole-plant corn material was chopped into 2 cm at the farm and taken into the laboratory immediately. For each of the 3 locations, there were 36 piles of forage (1 untreated pile and 2 LAB inoculated piles for each ensiling time of 0.5, 1, 3, 7, 14, and 60 d and for two temperatures.). The corn piles from each location were then inoculated with distilled water (control, CK), Lactobacillus plantarum MTD/1 (LP, 1 × 10^5^ CFU/g fresh matter, Vita Plus, Madison, MI, USA) and Lactobacillus buchneri 40788 (LB, 1 × 10^5^ CFU/g fresh matter, Vita Plus, Madison, MI, US), separately. The silage bags were stored in a 30°C incubator and an ∼20°C ambient temperature room for 0.5, 1, 3, 7, 14, and 60 d.

### Fermentation and chemical profile analyses.

After opening 0.5, 1, 3, 7, 14, and 60 d silage bags, 20 g of fresh and silage sample was homogenized with 180 mL distilled water in a juice extractor at a high speed for 30 s, and then filtrated through four layers of medical gauze. After measurement of pH using a glass electrode pH meter (PHSJ-3F, CANY, Shanghai, China), an aliquot of the filtrate was acidified with H_2_SO_4_ (7.14 M) and filtered with a 0.22-mm dialyzer for analysis of organic acid concentrations using High Performance Liquid Chromatography system (HPLC, KC-811 column, Shodex, Shimadzu, Japan; oven temperature: 50°C; flow rate: 1 mL/min; SPD: 210 nm; 33). Another aliquot of filtrate was used to determined WSC, ammonia nitrogen (NH_3_-N) and nonprotein nitrogen (NPN) (32, 33). Silage samples were dried at 55°C for 72 h for DM content measurement, and then ground with a mill for chemical composition analyses. The aNDF, ADF, and CP concentrations were determined according to the method described by Zhang et al. (32).

### Microbial composition SMRT analyses.

The total genomic DNAs of surface bacteria of fresh forage and silage samples were extracted using DNA isolation kit (Tiangen, DP302-02, Tiangen, China). The quantity and quality of extracted DNAs were measured using a NanoDrop ND-2000 spectrophotometer (Thermo Fisher Scientific, Waltham, MA, USA), Qubit 3.0 Flurometer (Life Technologies, CA, USA) and agarose gel electrophoresis, respectively. The PCR amplication, PCR amplicons purification and quantification and amplicons sequencing were performed according to the method described by Liu et al. (34).

After the comparison with the SILVA v138 database using the rdp_classifier-2.2 software. The reads belonging to unclassified *Lactobacillus*, *Weissella*, *Leuconostoc*, and *Pedicoccus* were subjected to the best hit method to gain species level information using BLASTN software (blast-2.11.0+). Alpha diversity analysis (Shannon index) was calculated with QIIME (version 1.9.0). The stream graph used to show the bacterial community successions was calculated by the method reported by Smits et al. (35). Two types of co-occurrence networks were constructed in the present study. The mate-network clustered by sensitive OTUs was constructed according to the analytical methods described by Hartman et al. (36). The co-occurrence networks were constructed by using the WGCNA package based on the Spearman’s correlation matrices according to the methods reported by Qiu et al. (37). Bacterial function prediction was proof checked from the Kyoto Encyclopedia of Genes and Genomes (KEGG) database using phylogenetic investigation of communities by reconstruction of unobserved states (PICRUSt2 v2.3.0_b; https://github.com/picrust/picrust2), which predicts the functional abundance of samples based on the abundance of marker gene sequences in the sample (38). Heatmap analysis was performed to identify the correlation between the main metabolic pathway and bacterial species by calculating the Spearman correlation index.

### Statistical analysis.

Data on silage fermentation were analyzed using Statistical Package for Social Science (SPSS 21.0, Inc., Chicago, IL, USA) according to a 2 × 3 × 6 factorial treatment design (two inoculant treatments, two storage temperatures, and six ensiling times):

$$Y_{\mathrm{ijk}}=\mu +D_{\text{i}}+T_{j}+I_{k}+{\left(D\times T\right)}_{\mathrm{ij}}+{\left(D\times I\right)}_{\mathrm{ik}}+{\left(T\times I\right)}_{\text{jk}}+{\left(D\times T\times I\right)}_{\text{ijk}}+e_{\mathrm{ijk}},$$

where Y_ijk_ represents the fermentative characteristics of the ensiled forages; μ is the overall mean; D_i_ is the effect of different ensiling days (i = 1, 2); T_j_ is the effect of different temperatures (j = 1, 2); I_k_ is the effect of different inoculants (k = 1, 2, 3); (D × T)_ij_ is the effect of interaction between ensiling days and temperatures; (D × I)_ik_ is the effect of interaction between ensiling days and inoculants; (T × I)_jk_ is the effect of interaction between temperatures and inoculants; (D × T × I)_ijk_ is the effect of interaction among ensiling days, temperatures and inoculants; and e_ijk_ is the random error term.

Data on chemical composition of 60 d silage were analyzed using SPSS 21.0 according to a 2 × 3 factorial design with two storage temperatures and 3 inoculants:

$$Y_{\mathrm{ij}}=\mu +I_{\text{i}}+T_{j}+{\left(I\times T\right)}_{\text{ij}}+\varepsilon_{\mathrm{ij}}$$

where Y_ij_ represents the response variable, μ is the overall mean, I_i_ is the effect of inoculants, T_j_ is the effect of storage temperature, (I × T)_ij_ is the effect of the interaction between the inoculants and storage temperature, and ε_ij_ is the random residual error. Significance was considered at *P < *0.05.

### Data availability.

Raw sequencing files and associated metadata have been deposited in NCBI’s Sequence Read Archive (PRJNA782958).
